# Evaluation of the coracoid bone tunnel placement on Dog Bone™ button fixation for acromioclavicular joint dislocation: a cadaver study combined with finite element analysis

**DOI:** 10.1186/s12891-022-06119-6

**Published:** 2023-01-09

**Authors:** Rangshan Gao, Wendong Zhang, Yuxia Yang, Yucheng Zhang, Yangyang Hu, Honghai Wu, Mingsheng Liu, Wenyong Fei, Jingcheng Wang

**Affiliations:** 1 Dalian Medical University, Dalian, 116044 People’s Republic of China; 2 Sports Medicine Department, Northern Jiangsu People’s Hospital, Clinical Medical College, Yangzhou University, Yangzhou, 225001 People’s Republic of China

**Keywords:** Acromioclavicular joint dislocation, Dog bone™, Finite element analysis, Tunnel location, Cadaver study

## Abstract

**Background:**

Dog Bone™ button fixation is frequently used to treat acromioclavicular joint (ACJ) dislocation. However, various studies have reported complications after fixation.

**Objective:**

To investigate the effect of the coracoid bone tunnel location on the treatment of ACJ dislocation through single-tunnel coracoclavicular (CC) ligament fixation with the Dog Bone™ button.

**Methods:**

Six cadaveric shoulders were used. Each specimen was subjected to five testing conditions in the following order: (1) normal ACJ (Gn); (2) acromioclavicular and CC ligaments were removed (G0); (3) CC ligament reconstruction was performed using the Dog Bone™ technique, and the coracoid bone tunnel was at the center of the coracoid base (G1); (4) reconstruction was performed at 5 mm distal from the G1 site, along the axis of the coracoid (G2); (5) reconstruction was performed at 10 mm distal from the G1 site, along the axis of the coracoid (G3). The angles of pronation and supination of the clavicle under the same load (30 N) were measured. Next, a finite element (FE) model was created using computed tomography (CT) images of the normal shoulder. Model 1 (M1), model 2 (M2), and model 3 (M3) correspond to G1, G2, and G3, respectively. A force of 70 N was applied as a vertical upward load to the distal clavicle. Subsequently, the von Mises stress, the strain LE along the FiberWire, and the displacement nephogram of the three models were obtained.

**Results:**

After single-tunnel CC ligament fixation using the Dog Bone™ technique, the clavicle in the G2 group (20.50 (19.50, 21.25) °, 20.00 (18.75, 21.25) °) had the best rotational stability. The peak von Mises stress, the strain LE along the FiberWire, and the maximum displacement were smaller in M2 than in M1 and M3.

**Conclusions:**

When the coracoid bone tunnel was located 5 mm anterior to the center of the coracoid base (along the axis of the coracoid), the clavicle showed greater rotational stability.

## Background

An acromioclavicular joint (ACJ) dislocation is a common shoulder injury, which accounts for approximately 12% of all shoulder dislocations. The treatment options depend upon the degree of the ACJ dislocation. Indeed, a Rockwood type IV-VI dislocation is usually treated with surgery. However, surgical intervention for a Rockwood type III dislocation remains a widely discussed topic of debate, previous literature suggests that surgical treatment can be considered for patients who undertake a high demand for activity, such as young competitive athletes [1–4]. A wide variety of surgical techniques has been described for the surgical treatment of ACJ dislocations, with many focusing on the reconstruction of the injured coracoclavicular (CC) ligaments [5].

The single-tunnel CC ligaments fixation of a dislocated ACJ using the Dog Bone™ button represents a current clinical method of treatment. This technique aims to restore the level of strength and stability similar to the original anatomy, which will allow the patients to achieve painless shoulder movement without any restriction of movement [6]. However, previous clinical studies have reported complication rates from CC ligament reconstruction techniques ranging from 23 to 80% [5, 7–9]. The CC ligament reconstruction using a coracoid bone tunnel technique presents visualization challenges and a high degree of difficulty, leading to reports detailing that those surgeons performing this technique undergo ‘a steep learning curve’. Therefore, it is not surprising that iatrogenic complications, such as coracoid and clavicle fractures, occur from drilling in the coracoid [5]. However, relatively few studies exist that have attempted to identify the ideal tunnel location in the coracoid for the Dog Bone™ button fixation. One biomechanical study using synthetic bone models demonstrated that the risk of a coracoid fracture decreases following the placement of holes at the coracoid base [10]. Further work using computer simulations has shown that drilling at the base of the coracoid may reduce the risk of an intraoperative coracoid fracture compared to drilling at other locations in the coracoid [11]. However, they did not take into account the rotational stability of the clavicles.

To our knowledge, there is currently limited evaluation of the effect of the coracoid bone tunnel location on the rotational stability of the clavicle following a single-tunnel reconstruction of the CC ligament using Dog Bone™ buttons. Therefore, this experiment was established to investigate the relationship between them. Our hypothesis was that the rotational stability of the clavicle may not be the best when the coracoid bone tunnel is located at the center of the coracoid base.

To achieve these aims, this study implemented cadaver studies and finite element analysis (FEA), which is a branch of biomechanical research commonly used in engineering and dentistry. Moreover, FEA is currently widely used in medical research owing to its biomechanical properties as a non-invasive, low-cost, and high validity method.

## Materials and methods

### Cadaveric study

#### Specimen preparation

Six cadaver shoulders were used (two males and one female, all of whom died of non-orthopedic causes; mean age of 53.3 years; ranging from 45 to 62 years). All soft tissues of the ACJ in the cadavers were stripped and the ACJ anatomy was observed. The specimens were kept moist throughout the experiment via a 0.9% normal saline [5]. These cadavers used in this study were provided by the Clinical Medical College, Yangzhou University.

#### Coracoid and clavicle tunnel location for the dog bone™ fixation

**Fig. 1:**
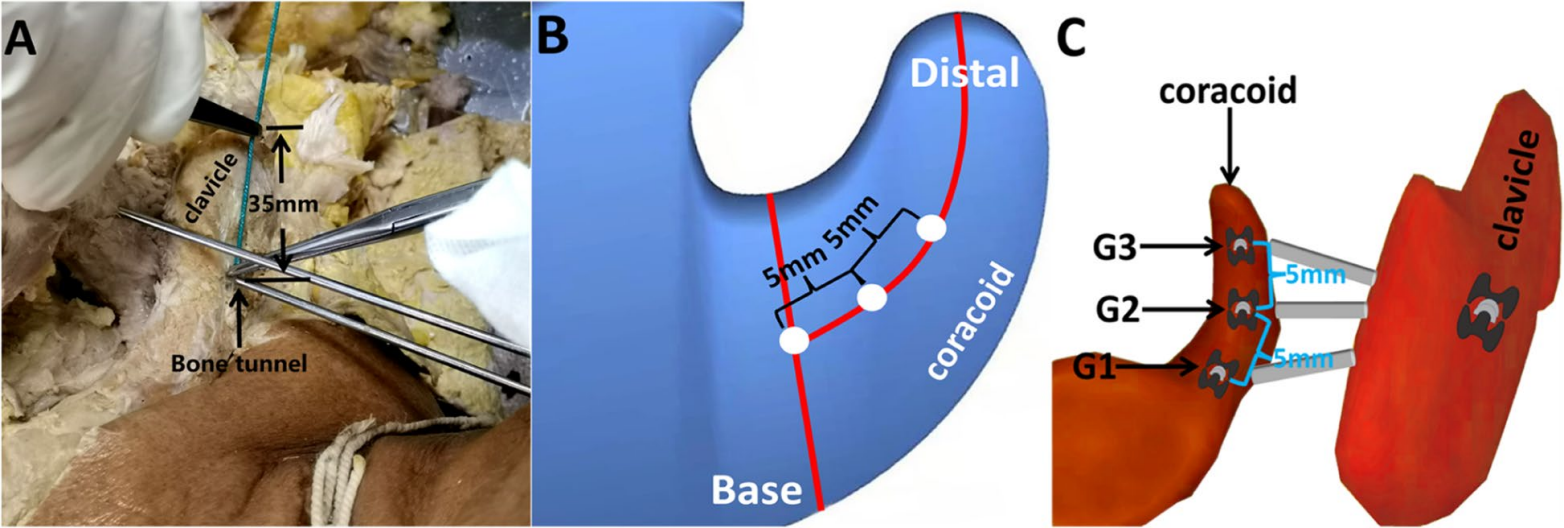
The location of the clavicle bone tunnel (**A**). Pattern diagram of the coracoid bone tunnel location. The white dots represent the locations of the coracoid bone tunnels (**B **&** C**)

The clavicle bone tunnel is located in the middle, approximately 35 mm from the distal end of the clavicle (Fig. 1A) [8, 12]. The middle of the coracoid base was identified by measuring the medial-lateral length of the base’s superior aspect with digital calipers. The midpoint was determined and used as the landmark for tunnel placement. The three drilling locations of the coracoid bone tunnel are: firstly, one is located centrally at the base of the coracoid (G1). The second is 5 mm distal along the long axis of the coracoid from the G1 site (G2), while the third is 10 mm distal along the long axis of the coracoid from the G1 site (G3) (Fig. 1B & C) [4, 5].

#### Single-tunnel fixation of the ACJ dislocation using the dog bone™ technique

**Fig. 2:**
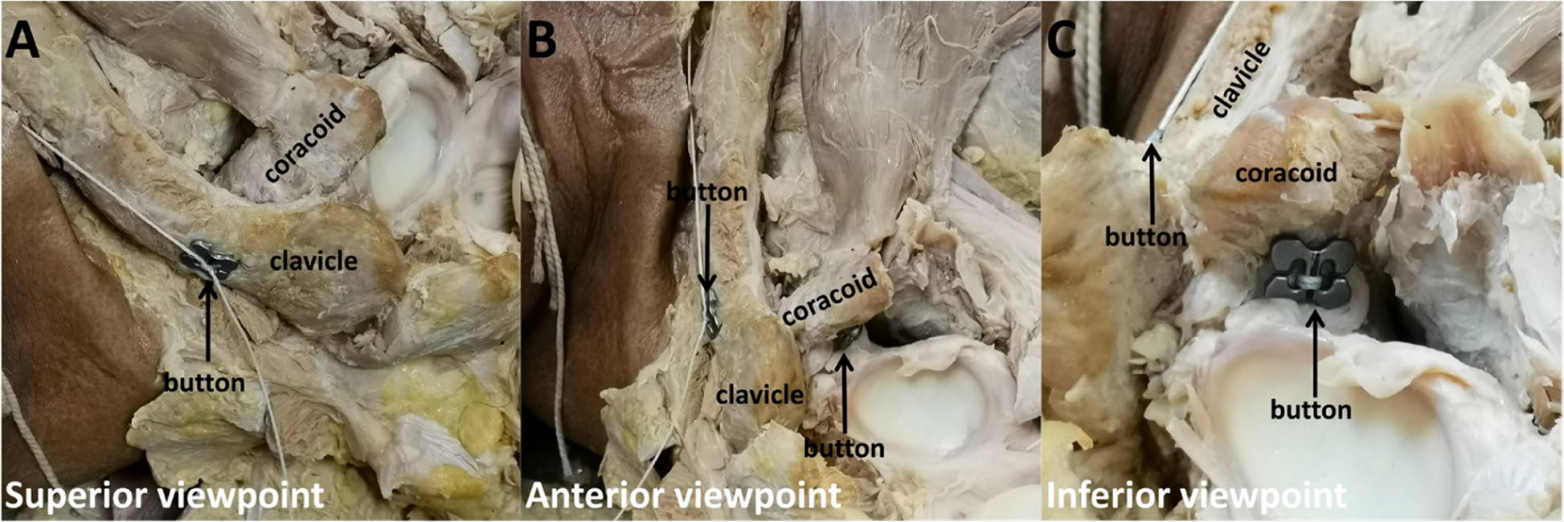
Various viewpoints of the Dog Bone™ buttons ACJ dislocation treatment. The pictures show the surgical technique from the superior viewpoint (**A**), anterior viewpoint (**B**), and inferior viewpoint (**C**)

In this experiment, the Burkhart-Dog Bone™ button single bundle reconstruction technique was implemented for the surgical reparation of the ACJ dislocations. This construct consists of 2 precontoured titanium cortical buttons connected by a FiberWire (Dog Bone™ button and FiberWire; Arthrex) (Fig. 2A, B & C).

#### Methods and indicators of the cadaveric study

To explore the rotational stability of the clavicle under different conditions, the cadaveric study was performed on each shoulder under five conditions. A normal ACJ (Gn), then in G0-G3, where the acromioclavicular and CC ligaments had been cut off to simulate a type III-V ACJ dislocation. Moreover, no buttons were used for the CC ligaments reconstruction (G0). In G1-G3, the CC ligament reconstruction was performed using the Dog Bone™ technique, whereby the clavicle bone tunnel was located in the middle site, approximately 35 mm from the distal end of the clavicle. However, the coracoid bone tunnel was either at the center of the coracoid base (G1), 5 mm distal from the G1 site, along the axis of the coracoid (G2), or 10 mm distal from the G1 site and along the axis of the coracoid (G3).

**Fig. 3:**
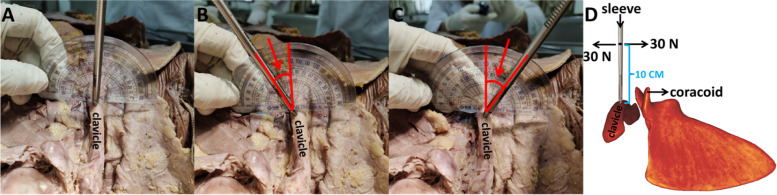
The angle of pronation (**A **&** B**) and supination (**A **&** C**) of the clavicle. Pattern diagram of the cadaveric study (**D**)

Firstly, a Kirschner wire with a 3 mm diameter was fixed in the middle of the clavicle. The Kirschner wire was then placed in a sleeve and a force-measuring device was used 10 cm from the top of the sleeve to apply a 30 N pulling force perpendicular to the sleeve and in the direction of the cadaver’s foot and head, respectively. The clavicle subsequently produced pronation and supination movements under the force. Finally, the angle of the Kirschner wire was measured using a goniometer (Fig. 3A, B, C & D).

### FE analysis

#### Three-dimensional geometry of the scapula and clavicle

**Fig. 4:**
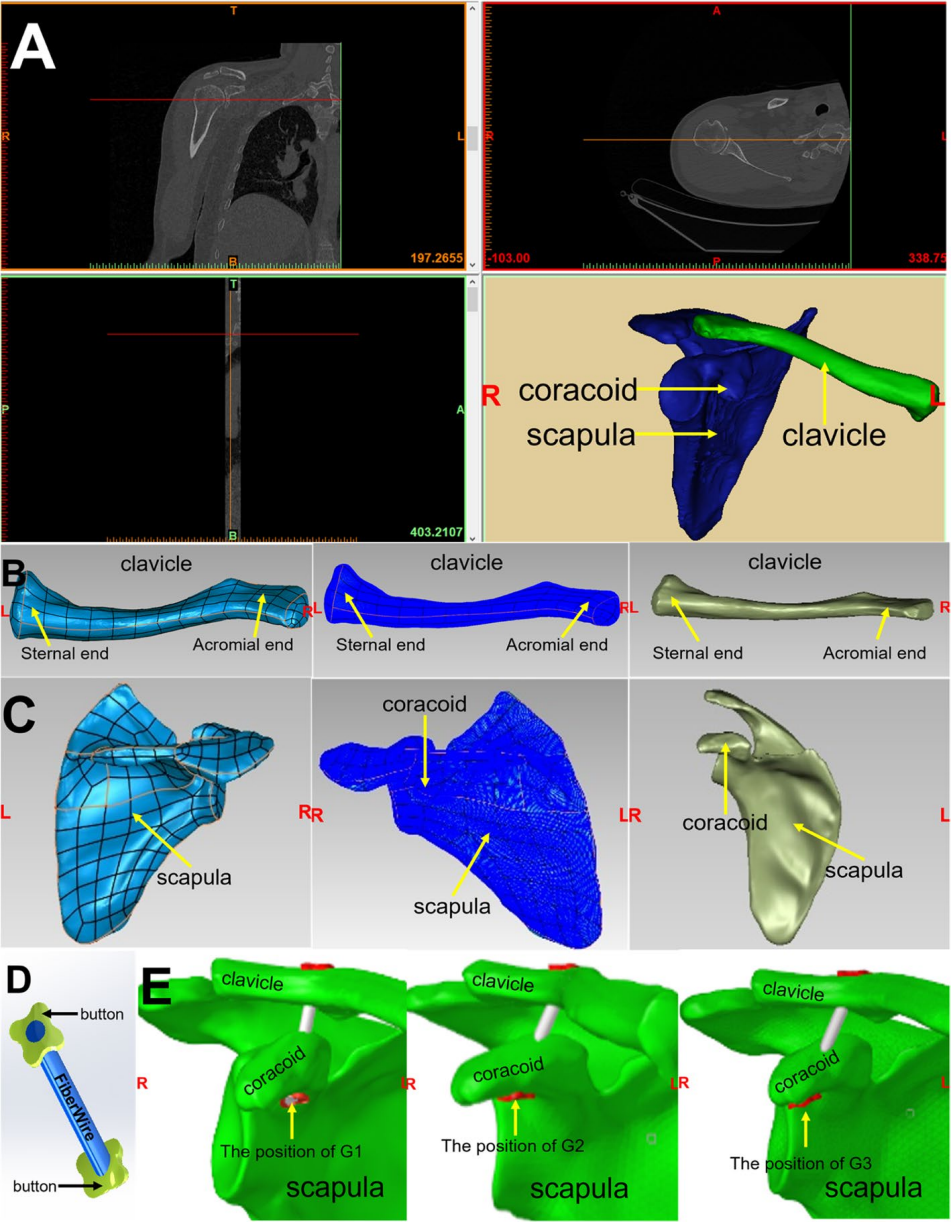
Image from CT scan (**A**). Process of modelling and meshing scapula (coracoid) and clavicle: based on a CT scan of the shoulder, a DICOM image was imported into Mimics software for 3D reconstruction of the scapula and clavicle. Geomatics software was used to fit the curved surface sheet, construct grids and generate solid geometry files (**B **&** C**). Dog Bone™ button with FiberWire: a simplified three-dimensional model of internal fixation of the Dog Bone™ technique (**D**). Dog Bone™ internal fixation models have been introduced into bone models to simulate CC ligament reconstruction at G1, G2, and G3, i.e., M1, M2, and M3 (**E**)

A healthy male volunteer (49 years old) was selected, and any clavicle fracture, scapula fracture, scapula deformity, and clavicle deformity were excluded. Informed consent was obtained, and the patient underwent computed tomography (CT) of the shoulder joint. Scanning parameters were as follows: scanning voltage 140 kV, scanning current 145 mA, matrix 512 × 512 px, layer thickness and layer spacing 1.25 mm. A total of 345 thin-layer images were obtained. The scapula and clavicle were modeled as follows:

- The thin-layer images were saved in DICM format, output by the PACS system, and imported into Mimics 19.0 software.
- After threshold adjustment, image mask segmentation, and other operations, 2D masks of the scapula and clavicle were obtained.
- Then, 3D reconstruction of 2D masks was carried out to obtain 3D models of the scapula and clavicle, which were exported as STL format files.
- In reverse surface operation, the STL model of the scapular and clavicle was imported into Geomagic Studio 2012 software, and the model was smoothed by filling the void and removing the features.
- Enter the precise surface module. After detecting the contour and constructing the curved surface and grid, the model was transformed into a solid geometry file, which was saved and exported in STEP format (to simplify calculations, all soft tissues were excluded) (Fig. 4A, B & C) [11, 13].

#### Three-dimensional models of the dog bone™ button and the FiberWire

Assemble the Dog Bone™ internal fixation models to the bone models:

- According to the parameters of the Dog Bone™ internal fixation models provided by the manufacturer, three-dimensional models of button internal fixation were drawn in SolidWorks software.
- The FiberWire was replaced by a cylinder with a diameter of 3 mm.
- The models were simplified appropriately (Fig. 4D) [3].

#### Three-dimensional model of the dog bone™ button for ACJ dislocation

- The geometric models of the four types of assembly (button; FiberWire; scapula and clavicle) were imported into HyperMesh 14.0 software, and mesh division and material attribute assignment were carried out.
- All parts were C3D4 units and imported into Abaqus 6.14 software in INP format.
- Based on cadaver studies, Dog Bone™ internal fixation models were introduced to bone models to simulate CC ligament reconstruction at G1 (drilling location of the coracoid bone tunnel located centrally at the base of the coracoid), G2 (5 mm distal along the long axis of the coracoid from the G1 site), and G3 (10 mm distal along the long axis of the coracoid from the G1 site), i.e., M1, M2, and M3 (Fig. 4E).

#### Material properties, load conditions, and boundary conditions

The material properties incorporated into this study were two independent parameters: Young’s modulus (E) and Poisson’s ratio (ν) (Table 1). All bones and implants were assumed to be homogeneous, isotropic, and linearly elastic. The biomechanical properties of bones, implants, buttons, and the FiberWire were applied to the models [6, 14, 15].

**Table 1:** Material properties of the models

Material	Young’s modulus (MPa)	Poisson’s ratio
Bone	17,000	0.30
Button	96,000	0.36
FiberWire	3800	0.28

*MPa* MegaPascals

**Fig. 5:**
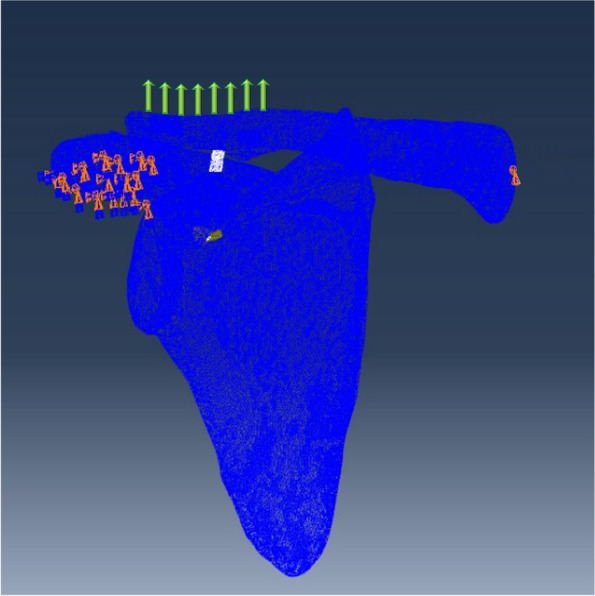
Boundary and loading conditions. Orange symbols indicate fixation points, and green arrows indicate applied force

**Fig. 6:**
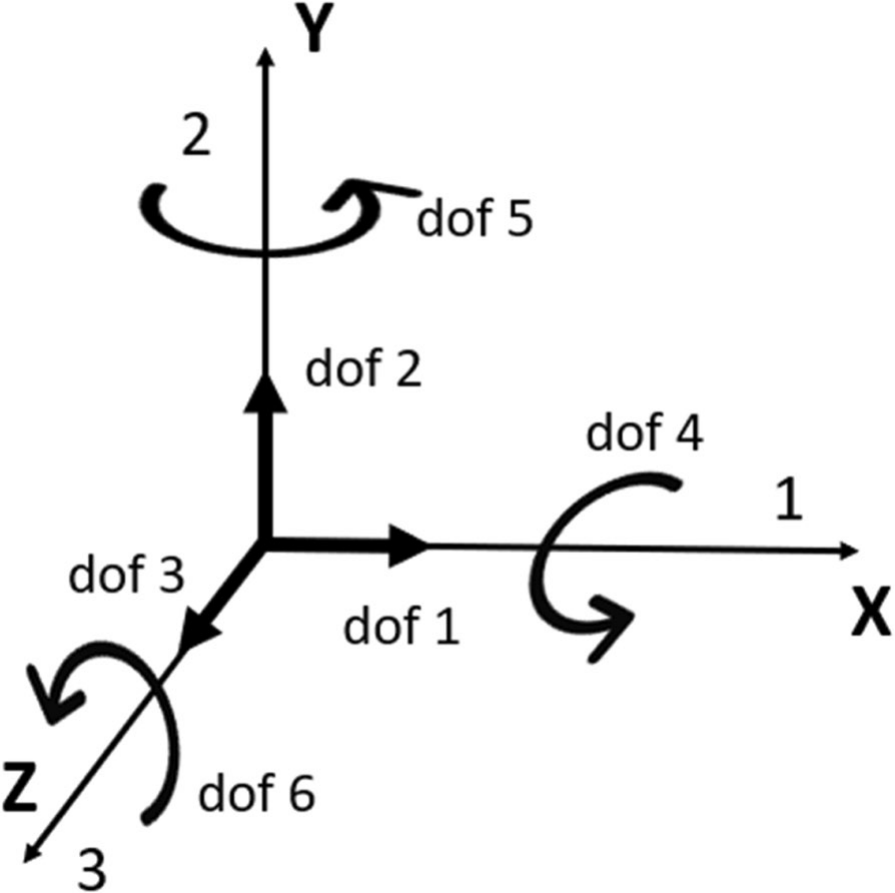
Schematic diagram of the degrees of freedom in Abaqus. Translation in the 1-direction (U1); translation in the 2-direction (U2); translation in the 3-direction (U3); rotation about the 1-direction (UR1); rotation about the 2-direction (UR2); rotation about the 3-direction (UR3). Dof: the degree of freedom

The load and boundary conditions used in the present study were derived from those used in a previous study. The sternal articular surface of the clavicle and the inferior surface of the acromion were determined for the boundary conditions (Fig. 5) [3, 13, 16, 17]. The FiberWire was bonded with buttons (TIE). Friction contact was established between the FiberWire, clavicular bone tunnel, and coracoid bone tunnel, and the contact factor was 0.46. Two buttons were subjected to frictional contact with the bone with a contact factor of 0.36. All degrees of freedom of the subacromial node were constrained to fix the scapula. The joints of the proximal clavicle were coupled to the center of the articular surface, constrained U1, U2, and U3 degrees of freedom of the center, and retained UR1, UR2, and UR3 degrees of freedom to achieve limited sliding of the proximal clavicle in the sternoclavicular joint (Fig. 6). The load condition was the external upward force (70 N) that acted on the distal clavicular area and simulated the stress of mild physical rehabilitation [18–20]. Finally, the displacement nephogram of the three models, the peak value of von Mises stress and the strain LE along the FiberWire were obtained.

#### Finite element analysis

Abaqus-CAE was used to build the finite element meshes with 4-noded linear tetrahedrons. The optimal number of elements was chosen after simulating the convergence analysis to obtain sufficient accuracy in the results, then all simulations were performed using Abaqus.

### Statistical analysis

Statistical analysis was performed by SPSS 26.0 (SPSS Inc., Chicago, IL) software. SW test was performed to determine whether the data was in accordance with the normal distribution. The data in accordance with the normal distribution was expressed as the mean ± standard deviation and data did not conform to the normal distribution was expressed as the median (25% quartile, 75% quartile). Kruskal-Wallis test was used to determine the difference among groups. The difference among groups was considered significant when *P* < 0.05.

## Results

### The cadaveric study

The results of the cadaveric study were summarized (Table 2). Pairwise comparison between Gn, G0, G1, G2 and G3 (Table 3). From Table 3, we can see that there was no significant difference between the G2 and Gn groups, while there was a significant difference between the G2 and G0 groups.

**Table 2:** Results of the cadaveric study

	Gn	G0	G1	G2	G3
Angles of pronation (°)	14.50 (12.75, 15.25)	48.00 (43.50, 52.00)	27.50 (25.50, 28.50)	20.50 (19.50, 21.25)	35.00 (32.75, 37.00)
Angles of supination (°)	14.50 (13.00, 15.25)	47.50 (42.75, 52.00)	28.00 (26.50, 30.00)	20.00 (18.75, 21.25)	36.00 (34.75, 36.50)

Data presented as the median (25% quartile, 75% quartile)

°Degree sign

**Table 3:** Pairwise comparison of pronation and supination angles between Gn, G0, G1, G2 and G3

	Gn-G2	Gn-G1	Gn-G3	Gn-G0	G1-G2	G2-G3	G0-G2	G1-G3	G0-G1	G0-G3
Pronation angles	*P* = 1.000	*P* = 0.181	*P* = 0.004^*^	*P* < 0.001^*^	*P* = 1.000	*P* = 0.181	*P* = 0.004^*^	*P* = 1.000	*P* = 0.181	*P* = 1.000
Supination angles	*P* = 1.000	*P* = 0.181	*P* = 0.004^*^	*P* < 0.001^*^	*P* = 1.000	*P* = 0.181	*P* = 0.004^*^	*P* = 1.000	*P* = 0.181	*P* = 1.000

^*^P-value is < 0.05

### The FEA

#### The displacement nephogram

**Fig. 7:**
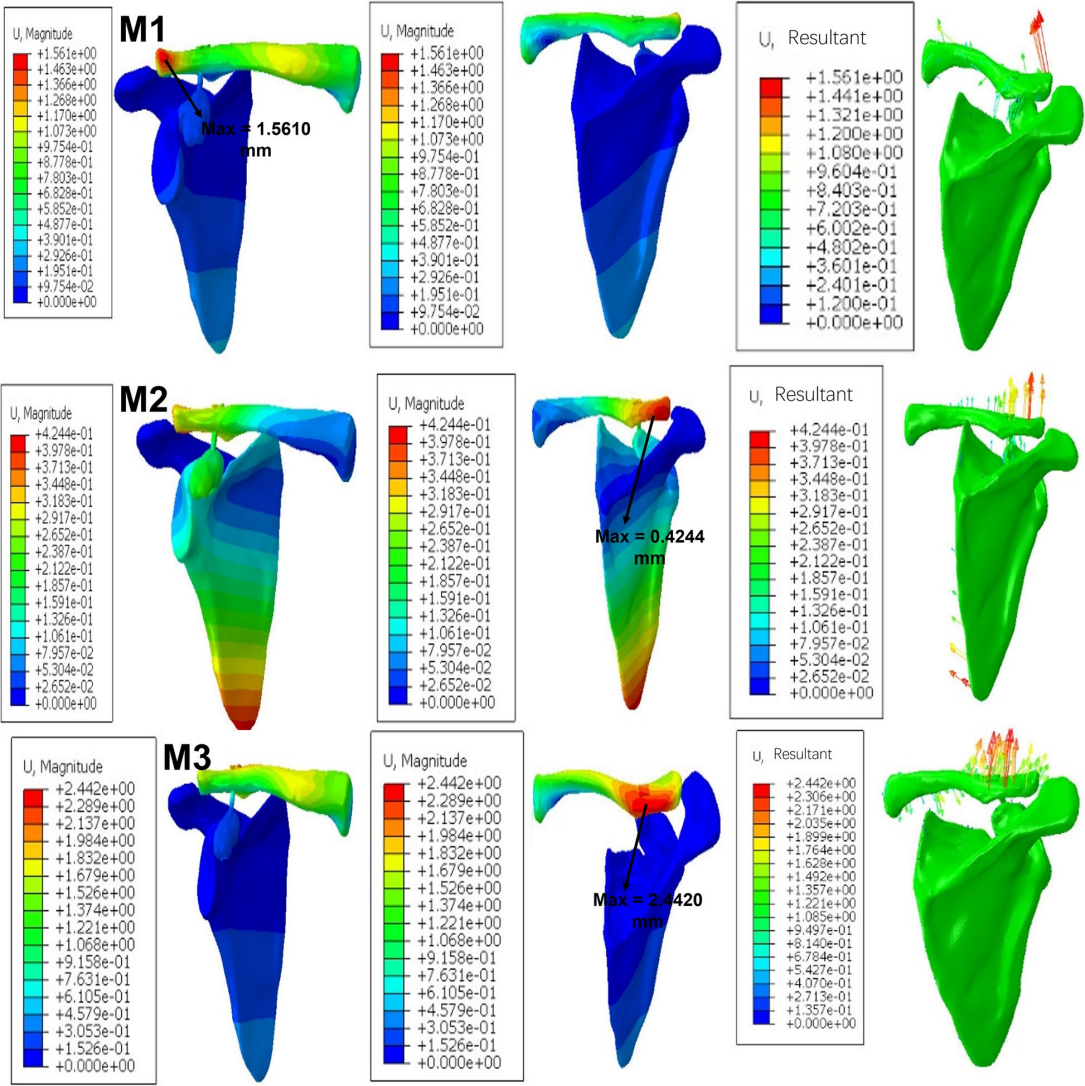
Displacement nephogram of M1, M2, and M3

Following the application of a loaded force to the distal end of the clavicle, the displacement nephogram of the FE models illustrated a maximum displacement of M1 located at the front of the clavicle’s distal end (1.5610 mm). The maximum displacement of M2 was located at the middle position of the distal end (0.4244 mm), while the maximum displacement of M3 was located behind the distal end (2.4420 mm) (Fig. 7).

#### The peak von Mises stress

**Fig. 8:**
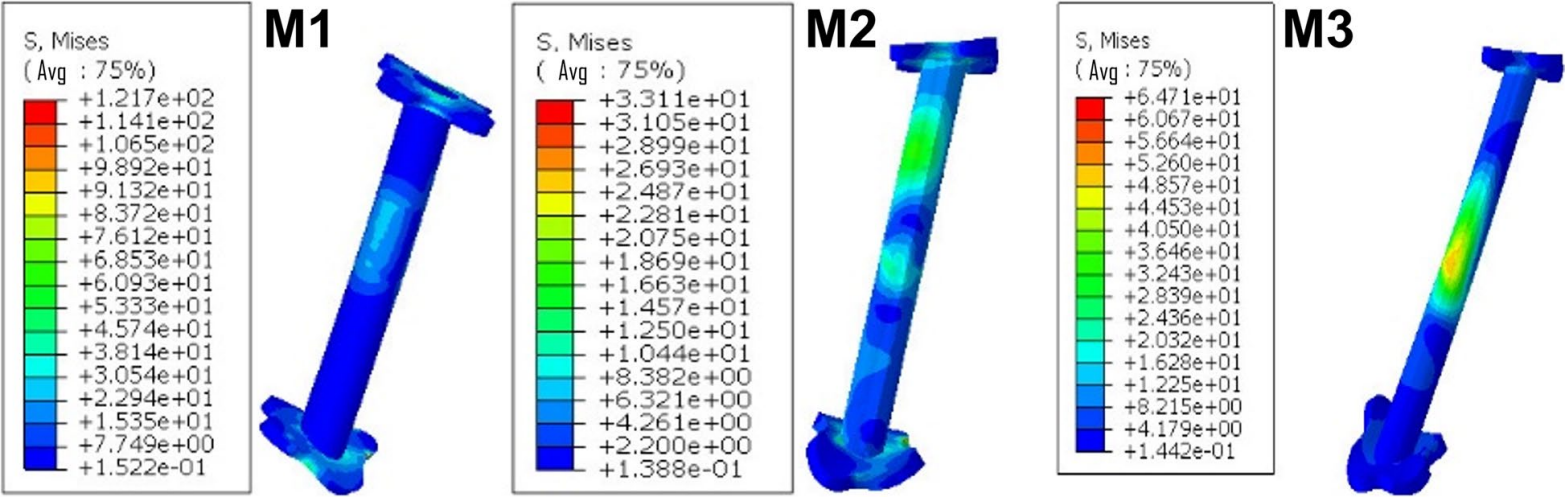
The peak value of the von Mises stress along the FiberWire in M1, M2, and M3

The peak value of the von Mises stress along the FiberWire in M1 was 121.70 MPa, while in M2 it was 33.11 MPa, and M3 produced 64.71 MPa (Fig. 8).

#### Strain LE

**Fig. 9:**
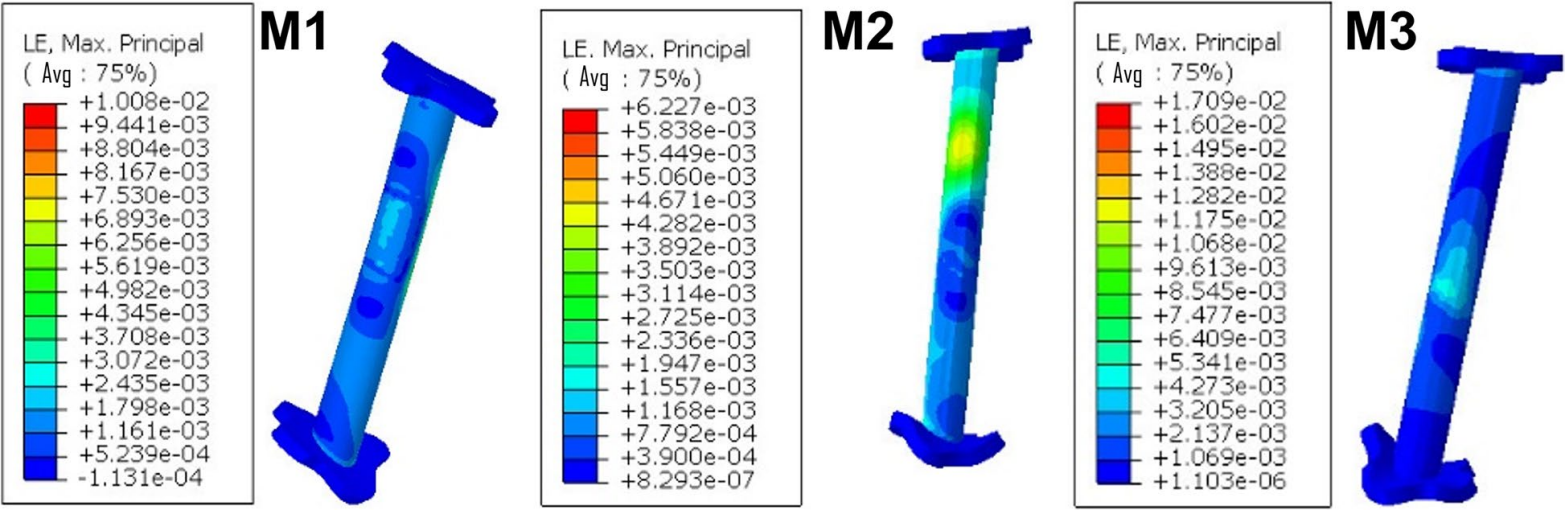
The strain LE along the FiberWire in M1, M2, and M3

The strain LE along the FiberWire in M1 was 0.010080, while in M2 it was 0.006227, and finally, in M3 it was 0.017090 (Fig. 9).

The loading values were summarized in Table 4.

**Table 4:** A summary of the FEA results

	Maximum displacements (mm)	The peak values of von Mises stress (MPa)	Strain LE
M1	1.5610	121.70	0.010080
M2	0.4244	33.11	0.006227
M3	2.4420	64.71	0.017090

*mm* Millimeter, *MPa* MegaPascals

## Discussion

This study aimed to investigate the effect the coracoid bone tunnel location had on the treatment of ACJ dislocation using a single-tunnel CC ligaments fixation with the Dog Bone™ button. Firstly, a cadaveric study was performed, followed by the implementation of FEA to further prove the validity of the cadaver study results. Overall, when the coracoid bone tunnel is located 5 mm anterior to the center of the coracoid base (along the axis of the coracoid), the clavicle gained greater rotational stability.

Previously, several postoperative complications have been reported following a single-tunnel CC ligament reconstruction of an ACJ dislocation using the Dog Bone™ technique. Shin et al. [21] reported a 33% rate of loss of reduction of more than 50% in 18 patients managed with a single-tunnel, adjustable-loop suspensory device. Cook et al. [22] reported that in 8 of 10 repairs (80%) intraoperative reduction was lost at an average of 7.0 weeks (range, 3–12 weeks), and four patients (40%) required revisions. Tunnel widening was universally noted, and the holding suture was prominently described as the failure mode in most patients. Dalos et al. [23] reported that tunnel widening was observed for Dog Bone™ technique and was located in the inferior parts of the clavicle and superior parts of the coracoid. However, the specific reason of postoperative tunnel widening remain unclear.

Based on the results of the cadaver study for G2, the angles of pronation and supination of the clavicle were 20.50 (19.50, 21.25) °, 20.00 (18.75, 21.25) °, respectively. There was no significant difference between the G2 and Gn groups; however, there was a significant difference between the G2 and G0 groups. For G1, there was no significant difference between the G1 and Gn groups, as well as the G1 and G0 groups. For G3, there was no significant difference between the G3 and G0 groups; however, there was a significant difference between the G3 and Gn groups. Therefore, the clavicle in the G2 group had better rotational stability in response to an external force.

Subsequently, FEA was implemented and the displacement nephogram of FE models shows that the maximum displacement of M1 was located in front of the distal end of the clavicle (1.5610 mm) and the clavicle tended to supinate. The location of M2 at the middle position of the distal end of the clavicle (0.4244 mm) and the displacement direction of the distal clavicle are almost vertical. Finally, M3 was located behind the distal end of the clavicle (2.4420 mm) and the clavicle tended to pronate. Since the coracoid bone tunnel is located in the center of the coracoid process base, it is relatively backward in M1. Thus, when the resultant force of the load was added to the distal clavicle it occurred in front of the distal clavicle, indicating that the displacement is greatest in front of the distal clavicle, which tends to supinate. For M2, the resultant force appears in the middle of the distal clavicle, so the displacement of the middle of the distal clavicle is the largest, making the distal clavicle move vertically. For M3, because the coracoid bone tunnel is located at the distal end of the coracoid process, its position is relatively forward. Therefore, the resultant force occurs behind the distal end of the clavicle, so the displacement behind the distal end of the clavicle is the largest and causes the tendency of the clavicle to pronate.

Generally, when the coracoid bone tunnel is located in the center of the base of the coracoid process or 10 mm anterior to the center of the base (along the axis of the coracoid), the action of external force promotes supination or pronation in the clavicle. This is consistent with the results of our cadaver study where the M2 clavicle has better rotational stability in response to an external force. Moreover, a supination or pronation movement in the clavicle causes the FiberWire to cut the clavicular and coracoid bone tunnel, which explains the postoperative tunnel widening. Hence, when the coracoid bone tunnel is located in the G1 site or 10 mm anterior to the G1 site (along the axis of the coracoid), the risk of a postoperative clavicular and coracoid fracture may be increased. Likewise, the erosion of clavicular and coracoid bone may be promoted by buttons and may also increase the risk of a clavicular or coracoid button failure. These complications may directly or indirectly lead to an ACJ dislocation reduction failure or re-dislocation.

In addition, the peak value of von Mises stress and the strain LE along the FiberWire were both smaller in M2 than in M1 and M3. This indicated that M2 has a stronger total construct.

Previous investigations have analyzed the location selection of the coracoid bone tunnel. Kummer et al. [10] used a combination of synthetic bone models and cadaveric scapulae to assess the effect of tunnels on the coracoid strength. Kummer and co-authors reported that the cadaveric specimens were more prone to fracture when tunnels were placed in the distal coracoid compared to the base. Campbell et al. [5] used six matched pairs of cadaveric scapulae to study the effect of the coracoid bone tunnel position on the treatment of an ACJ dislocation using Dog Bone™. They reported that the ultimate load for the centered tunnels in the distal coracoid absorbed a significantly higher ultimate load and energy compared to the eccentric tunnels. Although, they did not find a difference between the distal tunnels and the tunnels at the base. However, their study ignored the rotational stability of the clavicles. After research we found that: whereby during a single-tunnel reconstruction of the CC ligament, the different positions of the coracoid bone tunnel will lead to altering rotational stabilities of the clavicle. These will ultimately result in numerous cutting degrees of the FiberWire to the clavicle and coracoid bone tunnels. Indeed, these cuts may lead to either the clavicle and coracoid bone tunnels widening, which may potentially promote clavicular or coracoid fractures and other postoperative related complications. Therefore, to reduce the occurrence of these possible complications, our suggestion is that during a single-tunnel CC ligaments fixation of an ACJ dislocation using a Dog Bone™ button, the coracoid bone tunnel should be positioned 5 mm anterior to the center of the coracoid process base, along the axis of the coracoid. Although this would create a moment arm on the coracoid process, but a distance of 5 mm would not be too far from the center of the coracoid process base, which still provided considerable strength. Therefore, in general, we still recommended that the coracoid bone tunnel be located in 5 mm anterior to the center of the coracoid process base (along the axis of the coracoid).

As the research into ACJ dislocations increases, our current research aims are to explore a method that can both achieve ideal reduction and fixation alongside maximizing the recovery of ACJ mobility. Our results provide more information for orthopedic surgeons using a single-tunnel reconstruction of CC ligament with Dog Bone™.

### Deficiencies of the study

Several limitations within this study should be considered. Firstly, the use of cadaveric specimens during biomechanical testing does not accurately mimic the real situation in vivo with the various forces involved. Therefore, some degree of error is likely to occur [19]. Secondly, using cadaveric specimens for biomechanical testing does not provide information relating to biological healing. Consequently, result validation could not be performed on the long-term effects of the grafts on the clavicle and coracoid processes under different experimental conditions [24]. Thirdly, differences in scapula position can also change the relation between the clavicle and the coracoid. Is it possible that the rotational stability of the clavicle may be secondary to the angle between the line that intersects the insertion point in the clavicle and coracoid and the horizontal axis, for example? And thus, the further research in this field is needed. Finally, the application of the FEA in orthopedics was limited by the simplification of the model, whereby some of the more complex anatomies in the bone modeling phase was omitted. Therefore, the results obtained are often questioned [25]. The addition of the load to the models was only performed in a single direction, which was chosen because it accurately represented the clinical situation. However, in patients the loads applied to the construction would likely be multidirectional. It is possible that loads applied in other directions could lead to earlier or other causes of reduced ACJ dislocation failure and our conclusions cannot be directly applied to these situations [5].

### Future study

Our further studies will focus on clinical application of ACJ treatment using Dog Bone™ buttons. Their clinical effects will be investigated.

## Conclusions

When the coracoid bone tunnel was located 5 mm anterior to the center of the coracoid base (along the axis of the coracoid), the clavicle showed greater rotational stability.

## Data Availability

The datasets used and/or analyzed during the current study are available from the corresponding author on reasonable request.

## Abbreviations

ACJ: Acromioclavicular joint
CC: Coracoclavicular
FE: Finite element
FEA: Finite element analysis
CT: Computed tomography
